# The Methylation Capacity of Arsenic and Insulin Resistance are Associated with Psychological Characteristics in Children and Adolescents

**DOI:** 10.1038/s41598-017-03084-2

**Published:** 2017-06-08

**Authors:** Ying-Chin Lin, Chien-Tien Su, Horng-Sheng Shiue, Wei-Jen Chen, Yi-Hua Chen, Cheuk-Sing Choy, Hung-Yi Chiou, Bor-Cheng Han, Yu-Mei Hsueh

**Affiliations:** 1 Department of Family Medicine, Shung Ho Hospital, Taipei Medical University, Taipei, Taiwan; 2 Department of Health Examination, Wan Fang Hospital, Taipei Medical University, Taipei, Taiwan; 3 Department of Family Medicine, School of Medicine, College of Medicine, Taipei Medical University, Taipei, Taiwan; 4 Department of Family Medicine, Taipei Medical University Hospital, Taipei, Taiwan; 5 School of Public Health, College of Public Health, Taipei Medical University, Taipei, Taiwan; 6 Department of Chinese Medicine, Chang Gung Memorial Hospital, and Chang Gung University College of Medicine, Taoyuan, Taiwan; 7 Emergency Department, Taipei Hospital, Ministry of Health and Welfare, New Taipei City, Taiwan; 8 Department of Medicine, School of Medicine, College of Medicine, Taipei Medical University, Taipei, Taiwan; 9 Department of Public Health, School of Medicine, College of Medicine, Taipei Medical University, Taipei, Taiwan

## Abstract

The goal of the present study was to compare the influence of the methylation capacity of arsenic, as well as insulin resistance on psychological characteristics of school students from elementary and junior high school. 296 elementary and 318 junior high school students participated in health examinations, completed questionnaires and determined their concentrations of urinary arsenic species and psychological characteristics. Insulin resistance was determined by means of the Homeostatic Model Assessment of Insulin Resistance (HOMA-IR). We found that HOMA-IR values were significantly related to increased score of the depression and anger after adjusted for age, gender, schools, father’s educational levels, mother’s educational levels, BMI, body fat, and urinary creatinine in all students. Anxiety scores and depression scores of junior high school children were significantly higher compared to elementary school children, but lower in disruptive behavior scores. HOMA-IR levels were significantly inversely related to self-concept scores in junior high school students. A greater urinary inorganic arsenic percentage (iAs%) was marginally significantly related to a higher depression score in junior high school students. This is the first study to show a relationship between HOMA-IR levels or urinary arsenic profiles and psychological distress in school students from elementary and junior high school.

## Introduction

Arsenic is a naturally occurring metalloid, and is a known human carcinogen, promoting skin and lung cancer^1^. Epidemiological studies have documented neurotoxic effects in children with long-term arsenic exposure from contaminated milk powder^2^. Over the last decades, there has been an exponential increase in concern about the health risks of exposure to arsenic because of its potential neurotoxic effects^3^. Currently, the most concerning problem from a public health point of view is exposure to low doses of arsenic in children. Recently, low arsenic concentrations have been shown to be associated with an increased susceptibility to cognitive dysfunction^4^. In addition, a systematic review and meta-analysis study reported that arsenic exposure decreased children’s intelligence, measured by performing an intelligence quotient test^5^. Another review paper also highlighted that arsenic exposure increased the risk of impaired cognition and enhanced susceptibility for mood disorders in children^6^. Furthermore, another study provided additional evidence to support an association between arsenic exposure and impaired attention/cognitive functions, even at levels considered to be safe^7^.

The variability of arsenic methylation in the body may differentiate between the susceptibility to arsenic toxicity. Absorbed arsenate is reduced to arsenite and undergoes methylation to form monomethylarsonic acid (MMA^V^) and dimethylarsinic acid (DMA^V^), which have low toxicity^8^ and are excreted by the kidneys^9^. However, *in vitro* studies have suggested that intermediate metabolites of inorganic arsenic such as monomethylarsonous acid (MMA^III^) and dimethylarsenious acid (DMA^III^) are more toxic than inorganic arsenic^10,11^, although epidemiologic data are not available. Our previous prospective study found that, with age, the percentage of MMA^V^ (MMA^V^%) increased and the percentage of DMA^V^ (DMA^V^%) decreased significantly^12^, suggesting that a decrease in the methylation capacity of arsenic is associated with aging. Our recent study demonstrated that total urinary arsenic (sum of the inorganic and methylated arsenic species) is negatively associated with BMI in adolescents in Taiwan, and obese adolescents with high insulin levels had significantly higher MMA% and significantly lower DMA% compared to obese adolescents with low insulin^13^. This implies that obesity and high insulin levels were associated with a reduced methylation capacity of arsenic in adolescents. In addition, we also found the homeostasis model assessment of insulin resistance (HOMA-IR) value was significantly and positively related to total urinary arsenic concentrations and the body mass index (BMI) Z score. Higher BMI values and higher total urinary arsenic concentrations were associated with higher HOMA-IR values in children and adolescents in Taiwan^14^.

Overweight and obese children have been shown to have a high risk of developing physiological abnormalities^15,16^, as well as being prone to psychosocial distress including depression, anxiety and social withdrawal^17,18^, leading to a poor quality of life^19^, and behavioral problems^20,21^. A recent study reported that children who were overweight/obese had significantly lower self-concept and less disruptive behavior^22^. In addition, a recent study reported that a low mood/depression in healthy children was associated with high HOMA-IR levels^23^.

It remains to be determined whether urinary arsenic profiles and insulin resistance can influence psychosocial distress (depression, anger, self-concept, anxiety, and disruptive behavior) in adolescents, even with low arsenic exposure. Therefore, the goal of the present study was to explore the effect of arsenic exposure, and insulin resistance using the HOMA-IR index on psychosocial distress in elementary school and junior high school students in an area of Taiwan with low arsenic exposure.

## Results

Table 1 shows the city areas and schools in which children were living and studying. Fathers and mothers of elementary school students had higher educational levels than those of junior high school students. Among elementary school students, body fat percent, cholesterol, high-density lipoprotein (HDL), low-density lipoprotein (LDL), glutamate oxaloacetate transaminase (GOT), and urea levels were significantly higher compared to junior high school students, and blood sugar, serum insulin, HOMA-IR values, and urine creatinine were significantly lower (Table 1). The total urinary arsenic concentrations (μg/g creatinine) and DMA% were significantly higher in elementary school students compared to junior high school students, and iAs% was significantly lower. It seems that the methylation capability of arsenic in elementary school students was more efficient than that of junior school students (Table 1).

**Table 1:** Physiological characteristics and urinary arsenic profiles stratified by educational levels.

Variables	Elementary school students (N = 296)	Junior high school students (N = 318)	*p* value
Age (Mean ± SE)	8.84 ± 0.09	12.69 ± 0.03	<0.01
Gender			
Male	155 (52.36)	174 (54.72)	0.56
Female	141 (47.64)	144 (45.28)	
City areas			
Taipei City	117 (39.53)	318 (100)	
New Taipei City	179 (60.47)	—	
Schools			
San Xing Elementary School	41 (13.99)	—	
Wu Xing Elementary School	50 (17.06)	—	
Xin Yi Elementary School	23 (7.85)	—	
Ding Xi Elementary School	40 (13.65)	—	
Xin He Elementary School	5 (1.71)	—	
Shuang Cheng Elementary School	24 (8.19)	—	
Yong He Elementary School	38 (12.97)	—	
An Keng Elementary School	72 (24.57)	—	
Cheng De Junior High School	—	63 (19.81)	
Yon Ji Junior High School	—	255 (80.19)	
Father’s educational levels			
Illiterate	2 (0.71)	9 (3.00)	<0.01
Elementary School	20 (7.07)	36 (12.00)	
Junior high school	101 (35.69)	131 (43.67)	
Senior high school	133 (47.00)	109 (36.33)	
College and above	27 (9.54)	15 (5.00)	
Mother’s educational levels			
Illiterate	2 (0.70)	7 (2.25)	<0.01
Elementary School	13 (4.56)	39 (12.54)	
Junior high school	135 (47.37)	152 (48.87)	
Senior high school	123 (43.16)	105 (33.76)	
College and above	12 (4.21)	8 (2.57)	
Physiological characteristics			
BMI (kg/m^2^)	19.99 ± 0.26	20.56 ± 0.24	0.10
BMI group			
Lower than normal weight	36 (12.16)	52 (16.35)	<0.01
Normal Weight	108 (36.49)	168 (52.83)	
Overweight/obese	152 (51.35)	98 (30.82)	
Body fat (%)	26.18 ± 0.56	21.87 ± 0.45	<0.01
Cholesterol (mg/dL)	173.9 ± 1.72	160.1 ± 1.54	<0.01
Triglyceride (mg/dL)	71.65 ± 2.19	75.53 ± 2.22	0.21
HDL (mg/dL)	59.31 ± 0.71	55.82 ± 0.71	<0.01
LDL (mg/dL)	100.3 ± 1.46	90.03 ± 1.30	<0.01
GOT (IU/L)	25.51 ± 0.53	21.07 ± 0.45	<0.01
GPT (IU/L)	18.82 ± 1.15	16.20 ± 0.98	0.08
Blood glucose	88.92 ± 1.11	91.76 ± 0.41	0.02
Insulin (μIU/mL)	11.91 ± 1.07	16.81 ± 0.76	<0.01
HOMA-IR value	2.80 ± 0.32	3.92 ± 0.21	<0.01
Urea	12.17 ± 0.16	11.55 ± 0.15	<0.01
Urine creatinine (mg/L)	93.10 ± 2.71	128.7 ± 3.60	<0.01
Urinary arsenic indices			
Urinary total arsenic (μg/L)	24.60 ± 1.24	25.96 ± 1.24	0.44
Urinary total arsenic (μg/g creatinine)	29.94 ± 1.76	23.60 ± 1.35	<0.01
iAs%	4.78 ± 0.32	7.52 ± 0.31	<0.01
MMA%	5.01 ± 0.32	5.29 ± 0.28	0.51
DMA%	90.22 ± 0.52	87.20 ± 0.44	<0.01

Categorical and continuous variables are expressed as numbers (percentage) and mean ± Standard error, respectively.

HDL, high-density lipoprotein; LDL, low-density lipoprotein; GOT, glutamic oxaloacetic transaminase; GPT, glutamic pyruvic transaminase. HOMA-IR, Homeostasis model assessment of insulin resistance; HOMA-IR = Fasting insulin (μU/mL) × Fasting glucose (mg/dL)/405; iAs%: inorganic arsenic (iAs^III^ + iAs^V^)/total arsenic × 100; MMA%: MMA/total arsenic × 100; DMA%: DMA/total arsenic × 100.

The data regarding schooling was unavailable for three children; educational levels were unavailable for 31 children’ fathers and 18 children’ mothers.

Table 2 shows that anxiety scores and depression scores of junior high school children were significantly higher than those of elementary school children. In contrast, disruptive behavior scores of junior high school children were significantly lower than those of elementary school children. The distribution of categorized strata of anxiety scores and depression scores in junior high school was significantly different from those in elementary school children (Table 2).

**Table 2:** Psychological characteristics stratified by educational levels.

Variables	Elementary school students (N = 296)	Junior high school students (N = 318)	*p* value
Self-concept	50.58 ± 0.44	50.50 ± 0.46	0.91
Higher than normal	75 (25.34)	82 (25.79)	0.14
Normal	169 (57.09)	161 (50.63)	
Lower than normal	52 (17.57)	75 (23.58)	
Anxiety	46.96 ± 0.48	49.95 ± 0.58	<0.01
Normal	255 (86.15)	220 (69.18)	<0.01
Mild	20 (6.76)	43 (13.52)	
Moderate/Severe	21 (7.09)	55 (17.30)	
Depression	45.42 ± 0.52	47.70 ± 0.57	<0.01
Normal	257 (86.82)	243 (76.42)	<0.01
Mild	18 (6.08)	36 (11.32)	
Moderate/Severe	21 (7.09)	39 (12.26)	
Anger	46.94 ± 0.51	46.80 ± 0.57	0.86
Normal	249 (84.12)	258 (81.13)	0.57
Mild	24 (8.11)	33 (10.38)	
Moderate/Severe	23 (7.77)	27 (8.49)	
Disruptive behavior	46.82 ± 0.49	44.54 ± 0.54	<0.01
Normal	249 (84.12)	264 (83.02)	0.69
Mild	28 (9.46)	28 (8.81)	
Moderate/Severe	19 (6.42)	26 (8.18)	

The comparison of total urinary arsenic concentrations, arsenic methylation indices and HOMA-IR values between different psychological characteristics groups in elementary school students is presented in Table 3. Total urinary arsenic concentration in students with a mild depression score was significantly higher than students with a normal and moderate/severe depression score in elementary school students. In addition, Total urinary arsenic concentration in students with a mild depression score and moderate/severe depression score was significantly higher than students with a normal depression score. Regarding arsenic methylation indices, we found that iAs% was significantly lower, and DMA% was significantly higher in students with abnormal depression score and anxiety score than in students with a normal depression score and anxiety score.

**Table 3:** Distribution of urinary total arsenic and HOMA-IR values according to psychological characteristics in elementary school students.

Variables	N	Urinary total arsenic (μg/L)	Urinary total arsenic (μg/g creatinine)	iAs%	MMA%	DMA%	HOMA-IR value
Self-concept							
Higher than normal	75	25.87 ± 2.78	34.57 ± 4.71	5.61 ± 0.92	5.79 ± 0.83	88.60 ± 1.56	3.03 ± 0.79
Normal	169	24.30 ± 1.62	27.28 ± 1.86	4.68 ± 0.35	4.42 ± 0.35	90.80 ± 0.52	2.48 ± 0.26
Lower than normal	52	23.77 ± 2.44	31.90 ± 4.13	3.90 ± 0.38	5.45 ± 0.81	90.65 ± 0.98	3.53 ± 1.12
*p* value		0.83	0.19	0.20	0.21	0.20	0.44
Normal	169	24.30 ± 1.62	27.28 ± 1.86	4.68 ± 0.35	4.52 ± 0.35	90.80 ± 0.52	2.48 ± 0.26
Abnormal	127	25.01 ± 1.92	33.48 ± 3.24	4.91 ± 0.57	5.65 ± 0.59	89.44 ± 1.00	3.23 ± 0.66
*p* value		0.78	0.10	0.73	0.10	0.23	0.28
Anxiety							
Normal	255	23.82 ± 1.24	29.07 ± 1.83	5.03 ± 0.35	5.20 ± 0.36	89.77 ± 0.58	2.59 ± 0.33
Mild	20	28.20 ± 8.24	32.33 ± 9.66	2.42 ± 0.43	3.91 ± 1.40	93.67 ± 1.42	4.89 ± 1.05
Moderate/Severe	21	30.71 ± 3.94	38.25 ± 6.23	3.96 ± 0.93	3.67 ± 0.62	92.38 ± 1.24	3.31 ± 1.62
*p* value		0.27	0.38	0.09	0.32	0.09	0.17
Normal	255	23.82 ± 1.24	29.07 ± 1.83	5.03 ± 0.35	5.20 ± 0.36	89.77 ± 0.58	2.59 ± 0.33
Abnormal	41	29.49 ± 4.44	35.36 ± 5.64	3.21 ± 0.53	3.79 ± 0.74	93.01 ± 0.93	4.08 ± 0.97
*p* value		0.23	0.22	<0.01	0.13	<0.01	0.10
Depression							
Normal	257	23.59 ± 1.16^c^	29.25 ± 1.72	4.95 ± 0.36	5.22 ± 0.36	89.83 ± 0.58	2.80 ± 0.36
Mild	18	39.86 ± 10.54^c,d^	44.01 ± 13.72	3.42 ± 0.55	4.56 ± 1.42	92.02 ± 1.49	2.96 ± 0.79
Moderate/Severe	21	23.96 ± 4.15^d^	26.36 ± 5.50	3.84 ± 0.79	2.77 ± 0.58	93.39 ± 0.94	2.65 ± 0.65
*p* value		<0.01	0.11	0.37	0.15	0.15	0.98
Normal	257	23.59 ± 1.16^a^	29.25 ± 1.72	4.95 ± 0.36	5.22 ± 0.36	89.83 ± 0.58	2.80 ± 0.36
Abnormal	39	31.29 ± 5.43	34.50 ± 7.04	3.65 ± 0.49	3.60 ± 0.73	92.76 ± 0.85	2.79 ± 0.50
*p* value		0.04	0.47	0.03	0.09	<0.01	0.99
Anger							
Normal	249	23.80 ± 1.31	29.74 ± 1.86	4.83 ± 0.36	4.99 ± 0.35	90.18 ± 0.59	2.73 ± 0.30
Mild	24	26.27 ± 4.08	28.32 ± 5.10	4.81 ± 1.03	5.14 ± 1.12	90.05 ± 1.51	3.64 ± 2.41
Moderate/Severe	23	31.52 ± 5.79	33.78 ± 8.97	4.23 ± 0.65	5.03 ± 1.36	90.74 ± 1.45	2.64 ± 0.56
*p* value		0.23	0.80	0.88	0.99	0.96	0.73
Normal	249	23.80 ± 1.31	29.74 ± 1.86	4.83 ± 0.36	4.99 ± 0.35	90.18 ± 0.59	2.73 ± 0.30
Abnormal	47	28.83 ± 3.50	30.99 ± 5.06	4.52 ± 0.61	5.09 ± 0.87	90.39 ± 1.04	3.15 ± 1.25
*p* value		0.14	0.80	0.67	0.92	0.86	0.75
Disruptive behavior							
Normal	249	24.35 ± 1.33	29.71 ± 1.87	4.67 ± 0.36	5.10 ± 0.37	90.23 ± 0.60	2.62 ± 0.29
Mild	28	25.37 ± 5.14	33.12 ± 7.86	5.15 ± 0.83	3.51 ± 0.58	91.34 ± 1.20	2.90 ± 0.67
Moderate/Severe	19	26.77 ± 3.74	28.29 ± 4.53	5.60 ± 0.77	5.98 ± 1.05	88.43 ± 1.27	5.04 ± 3.04
*p* value		0.88	0.83	0.72	0.27	0.55	0.17
Normal	249	24.35 ± 1.33	29.71 ± 1.87	4.67 ± 0.36	5.10 ± 0.37	90.23 ± 0.60	2.62 ± 0.29
Abnormal	47	25.94 ± 3.38	31.17 ± 5.00	5.33 ± 0.58	4.51 ± 0.57	90.16 ± 0.90	3.77 ± 1.28
*p* value		0.64	0.76	0.34	0.39	0.96	0.39

^c^Normal depression vs. Mild depression, *p* < 0.05; ^d^Mild depression vs. Moderate or Severe depression, *p* < 0.05.

The HOMA-IR value in students had a normal score or higher than normal score for self-concept were significantly lower than students with a lower than normal self-concept score among junior high school students. In addition, the HOMA-IR value in students with a mild depression score was significantly higher than students with a normal or moderate/severe depression score in junior high school students. On the other hand, iAs% in students with an abnormal depression score or abnormal disruptive behavior score was significantly higher than students with a normal depression score or normal disruptive behavior score in junior high school students (Table 4).

**Table 4:** Distribution of urinary total arsenic and HOMA-IR values according to psychological characteristics in junior high school students.

Variables	N	Urinary total arsenic (μg/L)	Urinary total arsenic (μg/g creatinine)	iAs%	MMA%	DMA%	HOMA-IR value
Self-concept							
Higher than normal	82	26.81 ± 2.23	23.66 ± 1.90	6.87 ± 0.51	5.55 ± 0.49	87.57 ± 0.82	3.54 ± 0.19^a^
Normal	161	26.84 ± 1.99	24.41 ± 2.24	7.99 ± 0.45	5.48 ± 0.44	86.53 ± 0.63	3.67 ± 0.21^b^
Lower than normal	75	23.13 ± 1.82	21.80 ± 2.27	7.21 ± 0.67	4.58 ± 0.46	88.21 ± 0.89	4.88 ± 0.71^a,b^
*p* value		0.45	0.74	0.27	0.37	0.27	0.03
Normal	161	26.84 ± 1.99	24.41 ± 2.24	7.99 ± 0.45	5.48 ± 0.44	86.53 ± 0.63	3.67 ± 0.21
Abnormal	157	25.05 ± 1.46	22.77 ± 1.47	7.03 ± 0.41	5.09 ± 0.34	87.88 ± 0.690	4.18 ± 0.36
*p* value		0.47	0.54	0.12	0.48	0.12	0.22
Anxiety							
Normal	220	25.25 ± 1.37	22.13 ± 1.54	7.24 ± 0.33	5.33 ± 0.33	87.43 ± 0.51	3.98 ± 0.26
Mild	43	31.29 ± 4.07	28.02 ± 4.33	8.66 ± 1.15	5.97 ± 0.80	85.37 ± 1.41	3.98 ± 0.58
Moderate/Severe	55	24.62 ± 3.28	26.03 ± 3.33	7.72 ± 0.76	4.58 ± 0.70	87.70 ± 1.01	3.64 ± 0.34
*p* value		0.23	0.24	0.28	0.38	0.25	0.82
Normal	220	25.25 ± 1.37	22.13 ± 1.54	7.24 ± 0.33	5.33 ± 0.33	87.43 ± 0.51	3.98 ± 0.26
Abnormal	98	27.54 ± 2.57	26.90 ± 2.65	8.13 ± 0.66	5.19 ± 0.53	86.68 ± 0.84	3.79 ± 0.32
*p* value		0.43	0.10	0.23	0.82	0.43	0.63
Depression							
Normal	243	25.43 ± 1.31	23.38 ± 1.53	7.04 ± 0.31^c^	5.29 ± 0.31	87.67 ± 0.47	3.77 ± 0.21^c^
Mild	36	33.04 ± 5.71	27.51 ± 5.08	9.44 ± 1.47^c^	5.73 ± 1.06	84.83 ± 1.75	5.81 ± 1.06^c,d^
Moderate/Severe	39	22.67 ± 2.66	21.40 ± 2.81	8.75 ± 0.67	4.84 ± 0.69	86.42 ± 1.13	3.13 ± 0.34^d^
*p* value		0.09	0.52	0.01	0.74	0.10	<0.01
Normal	243	25.43 ± 1.31	23.38 ± 1.53	7.04 ± 0.31	5.29 ± 0.31	87.67 ± 0.47	3.77 ± 0.21
Abnormal	75	27.65 ± 3.11	24.34 ± 2.84	9.08 ± 0.78	5.26 ± 0.62	85.66 ± 1.02	4.42 ± 0.56
*p* value		0.51	0.76	0.02	0.97	0.08	0.28
Anger							
Normal	258	25.87 ± 1.28	23.45 ± 1.49	7.45 ± 0.35	5.47 ± 0.32	87.08 ± 0.50	3.75 ± 0.18
Mild	33	32.44 ± 5.75	29.15 ± 5.09	6.99 ± 0.62	4.57 ± 0.64	88.44 ± 1.05	5.29 ± 1.32
Moderate/Severe	27	18.82 ± 3.35	18.31 ± 3.12	8.80 ± 0.98	4.43 ± 0.89	86.77 ± 1.59	3.86 ± 0.60
*p* value		0.06	0.21	0.40	0.40	0.62	0.08
Normal	258	25.87 ± 1.28	23.45 ± 1.49	7.45 ± 0.35	5.47 ± 0.32	87.08 ± 0.50	3.75 ± 0.18
Abnormal	60	26.31 ± 3.59	24.27 ± 3.19	7.81 ± 0.57	4.51 ± 0.53	87.69 ± 0.92	4.65 ± 0.77
*p* value		0.91	0.81	0.60	0.12	0.59	0.26
Disruptive behavior							
Normal	264	26.35 ± 1.35	23.86 ± 1.52	7.18 ± 0.33^c^	5.41 ± 0.32	87.41 ± 0.48	3.94 ± 0.22
Mild	28	22.34 ± 2.16	22.24 ± 3.24	9.73 ± 1.22^c^	4.65 ± 0.70	85.63 ± 1.53	4.63 ± 0.97
Moderate/Severe	26	25.83 ± 6.03	22.45 ± 4.72	8.53 ± 0.95	4.71 ± 0.80	86.75 ± 1.49	3.02 ± 0.30
*p* value		0.66	0.91	0.04	0.62	0.50	0.27
Normal	264	26.35 ± 1.35	23.86 ± 1.52	7.18 ± 0.33	5.41 ± 0.32	87.41 ± 0.48	3.94 ± 0.22
Abnormal	54	24.02 ± 3.09	22.34 ± 2.80	9.15 ± 0.78	4.68 ± 0.53	86.17 ± 1.06	3.85 ± 0.53
*p* value		0.48	0.67	0.02	0.24	0.29	0.88

aSelf-concept higher than normal vs. Self-concept lower than normal, *p* < 0.05; ^b^Normal self-concept vs. Self-concept lower than normal, *p* < 0.05; ^c^Normal depression vs. Mild depression, *p* < 0.05; ^d^Mild depression vs. Moderate or Severe depression, *p* < 0.05.

We explored the association between parents’ smoking status and the urinary total arsenic and arsenic methylation capacity indices of children (Supplementary Table S1); we found that the fathers’ smoking status only affected the MMA% of children in elementary school. However, MMA% was not related to psychological characteristics in elementary school students or junior high school students in this study; therefore, family smoking status would not act as a confounding factor for analyzing the relationship between urinary total arsenic, iAs%, DMA% and psychological characteristics in elementary school students or junior high school students. In addition, we compared the different status of body fat, vegetables and fruit intake, along with family history, on the HOMA-IR values in children (Supplementary Table S2). We found that body fat affected HOMA-IR values in both elementary and junior high students, and that the diabetes history of the mother only affected HOMA-IR values in junior high students.

A multiple linear regression analysis between urinary arsenic profile, HOMA-IR values and psychological characteristics is shown in Table 5. The HOMA-IR values were significantly positively related to the depression and anger score adjusted for age, gender, schools, father’s educational levels, mother’s educational levels, BMI, body fat, and urinary creatinine in all students. In junior high school students, the HOMA-IR values were significantly negatively related to the self-concept score, and positively correlated with the depression and anger score. In addition, urinary total arsenic concentration was significantly positively related to the self-concept score. Moreover, a greater iAs% was associated with a higher depression score, to a marginal degree. Conversely, iAs% values were significantly positively related to the self-concept score in elementary school students. In addition, DMA% values, and HOMA-IR values were positively related to the anxiety score in elementary school students.

**Table 5:** Multiple linear regression analyses of urinary arsenic profiles, HOMA-IR values and psychological characteristics.

Variables	Self-concept		Anxiety		Depression		Anger		Disruptive behavior	
	Model 1^a^ β (SE)	Model 2^b^ β (SE)	Model 1^a^ β (SE)	Model 2^b^ β (SE)	Model 1^a^ β (SE)	Model 2^b^ β (SE)	Model 1^a^ β (SE)	Model 2^b^ β (SE)	Model 1^a^ β (SE)	Model 2^b^ β (SE)
*Overall*										
Urinary total arsenic (μg/L)	0.025 (0.016)	0.024 (0.016)	0.014 (0.019)	0.015 (0.019)	−0.007 (0.019)	−0.003 (0.019)	−0.014 (0.019)	−0.011 (0.019)	0.003 (0.018)	0.005 (0.018)
iAs%	0.029 (0.060)		−0.007 (0.072)		0.075 (0.073)		0.058 (0.072)		0.062 (0.069)	
DMA%		−0.052 (0.039)		0.057 (0.047)		0.216 (0.109)		0.031 (0.047)		−0.0004 (0.046)
HOMA-IR value	−0.108 (0.089)	−0.105 (0.089)	0.170 (0.107)	0.166 (0.107)	0.219 (0.109)^*^	0.216 (0.109)^*^	0.211 (0.107)^*^	0.207 (0.107)^+^	0.076 (0.103)	0.074 (0.103)
*Elementary school students*										
Urinary total arsenic (μg/L)	0.006 (0.022)	0.009 (0.022)	−0.009 (0.025)	−0.012 (0.025)	−0.007 (0.027)	−0.009 (0.027)	−0.011 (0.26)	−0.012 (0.026)	−0.004 (0.093)	−0.005 (0.026)
iAs%	0.184 (0.079)^*^		−0.134 (0.090)		−0.067 (0.098)		−0.009 (0.095)		−0.049 (0.093)	
DMA%		−0.092 (0.049)^+^		0.127 (0.055)^*^		0.088 (0.060)		0.054 (0.058)		0.050 (0.057)
HOMA−IR value	0.087 (0.106)	0.091 (0.106)	0.247 (0.120)^*^	0.245 (0.120)^*^	0.196 (0.131)	0.196 (0.131)	0.104 (0.127)	0.106 (0.126)	0.097 (0.124)	0.096 (0.124)
*Junior high school students*										
Urinary total arsenic (μg/L)	0.046 (0.023)^*^	0.039 (0.022)	0.019 (0.029)	0.022 (0.029)	−0.021 (0.028)	−0.015 (0.028)	−0.022 (0.029)	−0.014 (0.028)	0.006 (0.027)	0.011 (0.027)
iAs%	−0.138 (0.088)		0.060 (0.115)		0.196 (0.111)^+^		0.100 (0.112)		0.136 (0.104)	
DMA%		0.004 (0.063)		0.002 (0.081)		−0.073 (0.079)		0.027 (0.079)		−0.029 (0.074)
HOMA-IR value	−0.373 (0.148)^*^	−0.358 (0.149)^*^	0.091 (0.193)	0.084 (0.193)	0.368 (0.187)^*^	0.356 (0.187)^+^	0.459 (0.188)^*^	0.444 (0.188)^*^	0.128 (0.175)	0.117 (0.176)

β, regression coefficient; SE, Standard error.

Multivariable linear regression analyses were adjusted for covariates, indicated age, gender, schools, father’s educational levels, mother’s educational levels. BMI, body fat, and urinary creatinine.

^a^Model 1: Psychological scores = β_0_ + β_1_·(Urinary total arsenic) + β_2_·(iAs%) + β_3_·(HOMA-IR value) + β_i_·(covariates).

^b^Model 2: Psychological scores = β_0_ + β_1_·(Urinary total arsenic) + β_2_·(DMA%) + β_3_·(HOMA-IR value) + β_i_·(covariates).

^+^0.05 ≤ *p* < 0.1. ^*^p < 0.05.

HOMA-IR value and iAs% were correlated with psychological characteristics respectively, therefore we analyzed the combined effects of HOMA-IR value and iAs% on psychological characteristics. We did not find any relationship between the combined effects of HOMA-IR values and iAs% and psychological characteristics in all students. Therefore, we found a sequential decrease in the OR of anxiety, depression and anger among those with no risk factor, one risk factor, and two risk factors (iAs% and HOMA-IR value) in a dose-dependent manner in elementary school students; inversely a sequential increase in the OR of depression among those with no risk factor, one risk factor, and two risk factors (iAs% and HOMA-IR value) in a dose-dependent manner in junior high school students (Table 6).

**Table 6:** The interaction of iAs% and HOMA-IR value on psychological characteristics.

Variables		Self-concept		Anxiety		Depression		Anger		Disruptive behavior	
		Normal/Abnormal	Multivariate ORs (95% CI)	Normal/Abnormal	Multivariate ORs (95% CI)	Normal/Abnormal	Multivariate ORs (95% CI)	Normal/Abnormal	Multivariate ORs (95% CI)	Normal/Abnormal	Multivariate ORs (95% CI)
*Overall*											
iAs%	HOMA-IR value										
≤5.18	≤2.42	92/78	1.00	141/29	1.00	146/24	1.00	139/31	1.00	142/28	1.00
≤5.18	>2.42	71/66	0.99 (0.58–1.69)	98/39	1.42 (0.75–2.71)	117/20	1.17 (0.56–2.46)	120/17	0.52 (024–1.11)	122/15	0.58 (0.26–1.28)
>5.18	≤2.42	78/59	0.81 (0.50–1.31)	107/30	0.91 (0.49–1.69)	111/26	1.25 (0.65–2.42)	110/27	0.93 (0.50–1.74)	114/23	0.92 (0.48–1.78)
>5.18	>2.42	89/81	0.99 (0.58–1.69)	129/41	0.76 (0.40–1.47)	126/44	1.58 (0.79–3.17)	138/32	0.68 (0.34–1.37)	135/35	1.09 (0.54–2.21)
*Elementary school students*											
iAs%	HOMA-IR value										
≤3.72	≤1.74	41/33	1.00	62/12	1.00^§^	63/11	1.00^§^	59/15	1.00^§^	64/10	1.00
≤3.72	>1.74	41/33	0.89 (0.41–1.90)	57/17	1.23 (0.46–3.31)	61/13	1.06 (0.36–3.11)	63/11	0.75 (0.26–2.18)	68/6	0.53 (0.16–1.77)
>3.72	≤1.74	47/27	0.70 (0.35–1.39)	65/9	0.49 (0.18–1.38)	64/10	0.70 (0.26–1.92)	60/14	0.78 (0.32–1.91)	58/16	1.69 (0.67–4.28)
>3.72	>1.74	40/34	1.00 (0.47–2.14)	71/3	0.16 (0.04–0.65)^*^	69/5	0.28 (0.08–1.02)^+^	67/7	0.21 (0.06–0.78)^*^	59/15	1.23 (0.43–3.51)
*Junior high school students*											
iAs%	HOMA-IR value										
≤6.49	≤3.02	39/39	1.00	56/22	1.00	67/11	1.00^§^	67/11	1.00	68/10	1.00
≤6.49	>3.02	37/44	1.43 (0.71–2.88)	51/30	1.67 (0.79-3.55)	65/16	1.89 (0.77–4.65)	68/13	1.20 (0.46–3.11)	72/9	0.87 (0.30–2.49)
>6.49	≤3.02	47/34	0.67 (0.34–1.30)	54/27	1.14 (0.55–2.34)	56/25	2.42 (1.06–5.52)^*^	61/20	1.87 (0.80–4.37)	61/20	2.03 (0.85–4.85)
>6.49	>3.02	38/40	1.41 (0.70–2.85)	59/19	0.81 (0.37–1.78)	55/23	2.29 (0.96–5.45)^+^	62/16	1.43 (0.57–3.59)	63/15	1.41 (0.54–3.70)

ORs, odds ratios; CI, 95% confidence intervals.

Multivariable logistic regression analyses were adjusted for covariates, indicated age, gender, schools, father’s educational levels, mother’s educational levels, BMI, body fat, and urinary creatinine.

^+^0.05 ≤ *p* < 0.1. ^*^p < 0.05. ^§^*p* < 0.05 for trend test.

## Discussion

To the best of our knowledge, this is the first study to show an association between multiple psychological characteristics including self-concept, anxiety, depression, anger, and disruptive behavior and HOMA-IR values and urinary arsenic profiles in children and adolescents in Taiwan. In this study, we have shown that elementary school students had a more efficient arsenic methylation capacity (higher DMA% and lower iAs%) than junior high school students; these results were similar to our previous study^13,14^. One interesting finding was that high HOMA-IR values were significantly associated with an increased depression and anger score in junior high school students and all students. A high HOMA-IR value was significantly associated with a lower self-concept score in junior high school students; in contrast, total urinary arsenic concentration was significantly related to the self-concept score in junior high school students. In addition, a higher iAs% was associated with a higher self-concept score in elementary school students and with a higher depression score in junior high school students after covariate adjustment.

The educational system in Taiwan is quite different from that in Western society; ninth grade (age 14–15) students have to face academic tracking in early adolescence^24^. A study reported that students taking the senior high school joint entrance examination slept less hours at night and were less alert during the daytime compared to those who were not taking the examination^25^, suggesting that the academic pressures that adolescents faced could influence their behavior. Therefore, the anxiety and depression scores were significantly higher in senior high school students than in the elementary school students of this study. By contrast, disruptive behavior scores of senior high school students were lower than those of elementary school students. A further study should be performed to verify this inconsistency.

Our previous study found that HOMA-IR values were significantly increased in relation to the total urinary arsenic (μg/L)^14^. This finding indicates that arsenic exposure may be related to β-cell dysfunction, increasing the risk of diabetes in Korean adults^26^. A longitudinal study found that healthy children with low moods had higher HOMA-IR values than those without^23^. This implies that neuropsychiatric syndromes alter metabolic networks such as insulin-glucose homeostasis, immuno-inflammatory processes and adipokine synthesis, and secretion is a defining pathophysiological component^27^. Understanding how depressive symptoms are linked to metabolism during childhood and adolescence may be important for identifying risk factors for diabetes. However, in our study, children with high HOMA-IR values were associated with high anger scores and depression scores. Taken together, high total urinary arsenic may induce an increase in HOMA-IR values, and result in impaired glucose homeostasis, which could be related to depression and anger in children and adolescents in Taiwan; however, this hypothesis needs further studies to confirm the underlying mechanisms of the reported association.

Insulin resistance has been shown to be negatively related to cognitive performance in adult humans^28^, and insulin resistance at the level of the brain may be associated with the effects on glucose uptake, and could influence mediators of brain function in adults^29^. However, one study reported that HOMA-IR was significantly and negatively correlated with self-perceived scholastic competence in girls^30^. We also found that HOMA-IR was significantly negatively associated with the self-concept score in junior high school students; the underlying reason why this relationship was age specific was beyond the scope of this study, but could be the subject of further investigation. By contrast, total urinary arsenic was significantly related to the self-concept score, which is opposite to what we originally hypothesized. This cannot be explained by the present study and needs further investigation. On the other hand, urinary arsenic concentrations greater than 50 μg/L were associated with poor scores on tests measuring visual-spatial reasoning, language and vocabulary, memory, intelligence, and math skills^31^, as well as a modest association with hyperactive behavior^32^ in 6–8 year-old children in Mexico. However, total urinary arsenic concentration was significantly related with the self-concept score in this study, which requires further investigation. However, an interesting result showed that iAs% was significantly related to increasing depression score in this study. This may suggest that arsenic depletes s-adenosylmethione (SAM)-methyl levels in the arsenic methylation pathway, leading to alterations in DNA methylation^33^, and this epigenetic modification to the DNA, which may result in aberrant gene expression, even in the brain^34^, increases the risk of impaired cognition and enhanced susceptibility for mood disorders. However, whether other arsenic methylation capacity indices can also influence psychological distress or the link between a particular gene of DNA methylation and cognition deficits has yet to be elucidated. Overall, arsenic methylation capacity leads to insulin resistance^35^, and may then disrupt the clearance of β-amyloid protein^36^; it may also increase tau dephosphorylation and microtubule binding of tau by weakening the activity of phosphoinositide 3-kinase/protein kinase B and adenosine monophosphate, resulting in neuronal degeneration^37^ related to cognitive dysfunction. Insulin resistance may be related to hypothalamic-pituitary adrenal axis dysfunction resulting in depressive symptoms^38^. Hippocampal neurogenesis reduction and increased advanced glycation end products were associated with depression^39^.

This study had several limitations that need to be taken into consideration when interpreting these results. Firstly, a single-spot measurement of urinary arsenic species and blood biochemical indices may not provide enough information. In addition, the methylation of arsenic and HOMA-IR values may be influenced by nutrients, for which information was unavailable in this study. However, the values could be reliable if all participants had no change to their lifestyle and maintained their homeostatic metabolism. Secondly, as this study had a cross-sectional design, it could not determine the causality of the observed associations. We cannot exclude the possibility that the association between high total urinary arsenic concentrations or high HOMA-IR values and psychological indices could be the result rather than the cause of a change in psychological index values. Thirdly, the measurement of psychological characteristics was self-reported using a rating scale, and may be incomplete or subject to socially desirable effects, interpretation of results should be treated with caution. Fourthly, we did not collect any information of potential factors, such as emission of exhaust gases, waste water or home decoration, which would probably affect arsenic methylation capacity. In addition, data concerning exercise was unavailable, and eating habits and family history include missing data; these variables may be related to insulin resistance in this study. The parents’ level of income, which may influence children’s psychological characteristics, was also unavailable. The fact that they cannot be adjusted is another limitation of this study. In addition, all students were sampled in Taipei city and New Taipei City (urban areas) and, therefore, our sample is a limited representation of the entire population of Taiwan. In spite of these limitations, this study represents the first attempt to address the effect of change in urinary arsenic profiles or HOMA-IR values on psychological characteristics after adjustments for BMI, educational levels and anthropometric measurements.

## Conclusion

This is the first study to show a relationship between HOMA-IR values or urinary arsenic profiles and psychological distress in children and adolescents with low arsenic exposure in Taiwan.

## Methods

### Study participants

Two cross-sectional studies were conducted. Eight elementary schools, including San Xing, Wu Xing, Xin Yi, Ding Xi, Xin He, Shuang Cheng, Yong He, and An Keng Elementary Schools in Taipei City or New Taipei City recruited 3,500 students in the first study, from September 2007 to September 2009. Ten percent of all elementary school students were randomly invited to attend Taipei Medical University Hospital for a detailed health examination. A total of 296 (84.57%) elementary school students volunteered to receive detailed health examinations, which were conducted at Taipei Medical University Hospital from September 2009 to December 2009^13^. Junior high school students from Cheng De and Yon Ji Junior High Schools in Taipei City took part in a second study, from October 2010 to November 2011, and recruited 318 students. The Research Ethics Committee of the Taipei Medical University, Taipei, Taiwan, approved the study, which was conducted in agreement with standards specified in the World Medical Association Declaration of Helsinki. All participants came from Taipei City or New Taipei City. All study participants provided either their parents’ or their own written informed consent form before participating in questionnaire interviews, or providing biological specimens. Two research assistants who had received 6 hours of specialized training performed the anthropometric measurements of weight and height for all elementary school students and junior high school students according to standard guidelines. Participants removed their shoes, and wore light clothing for measurements of standing height and weight in a rigid vertical position, using a standard medical balance scale. Height was measured to the nearest 0.5 cm, and weight was measured to the nearest 100 g. Body mass index (BMI) was calculated as weight (kg)/height (m^2^). Categories were divided into overweight, obese, and lower than normal weight, defined according to guidelines developed by the Ministry of Health and Welfare, Executive Yuan, Taiwan^40^ based on WHO Child Growth Standards^41^, and a modified locally weighted method^42^ designed for use with children and adolescents, based on BMI, age, and gender. Body fat as a percentage of weight was calculated, to obtain two measurements, by a commercially available bioelectrical impedance analyzer (Maltron BioScan 920 analyzer, Maltron International Ltd). All participants lived in Taipei City and New Taipei City, which are without obvious arsenic exposure. Participants drank tap water with arsenic levels less than the standard 10 μg/L provided by the Taipei Water Department of the Taipei City Government. The average arsenic concentration of tap water in Taipei City is 0.7 µg/L, and ranged from non-detectable to 4.0 µg/L.

### Questionnaire interview

Well-trained interviewers carried out face-to-face interviews to collect information using a structured questionnaire. The questionnaire included information on demographics and socioeconomic characteristics, lifestyle behavior of parents, such as cigarette smoking and alcohol consumption, and personal and family disease history.

### Biological specimen collection

About 5 to 8 mL of peripheral blood was collected from participants using vacuumed syringes at the time of recruitment. Blood samples were then separated into red blood cells and serum, and frozen at −80 °C for subsequently measuring biochemical indices. Concurrently, spot urine samples of 20 ml were collected and immediately transferred to a −20 °C freezer until required for urinary arsenic species analyses.

### Urinary arsenic species measurement

The analytical methods for determining urinary arsenic species have been described previously^43^. Briefly, urine samples were thawed at room temperature, ultrasonically mixed and filtered through a Sep-Pak C18 column. A 200-μL sample of treated urine was injected into a high-performance liquid chromatography column, linked to a hydride generator and atomic absorption spectrometer (HG-AAS) to measure the concentrations of arsenite (iAs^III^), arsenate (iAs^V^), MMA^V^ and DMA^V^. Recovery rates of the four arsenic species were calculated by (sample spiked standard solution concentration − sample concentration)/standard solution concentration × 100. The recovery rates ranged from 93.8% to 102.2%, and the detection limits for iAs^III^, DMA^V^, MMA^V^ and iAs^V^ were 0.02, 0.08, 0.05 and 0.07 μg/L respectively. The certified value of standard reference material (SRM 2670) was 480 ± 100 μg/L of inorganic arsenic obtained from the National Institute of Standards and Technology (NIST). SRM 2670 was used as a quality standard and analyzed along with urine samples. The mean value of SRM 2670 determined by our system was 507 ± 17 μg/L (n = 4). Both arsenobetain and arsenocholin are ingested from seafood and are excreted without metabolic transformation and, therefore, arsenobetain and arsenocholin were not measured by the HG-AAS method^44^.

### Serum biochemical examination

Total cholesterol, triglyceride, high density lipoprotein (HDL) cholesterol, low density lipoprotein (LDL) cholesterol and insulin serum levels were determined by means of an autoanalyzer (Hitachi 737, USA) with reagents obtained from Boehringer Mannheim Diagnostics. An enzymatic assay for serum homocysteine was described by Chan *et al*.^45^. A close correlation (r > 0.9) was observed between the results from the enzymatic method and a high performance liquid chromatography procedure used as ref.^45^. HOMA-IR values were calculated using the formula: fasting insulin (μU/mL) × fasting glucose (mg/dL)/405^46^.

### Measurement of psychological characteristics

We used the Beck Youth Inventories, second edition (BYI-II), to evaluate the children’s reported thoughts, feelings, and behavior related to emotional and social dysfunction^47^. A child’s experiences in the five psychological domains, namely self-concept (assessing cognitions of competence, potency, and positive self-worth), anxiety (assessing worries about school performance, the future, negative reactions of others, and fears), depression (assessing negative thoughts about the self, life, and the future, and feelings of sadness and guilt), anger (assessing thoughts of being treated unfairly by others, and feelings of anger and hatred), and disruptive behavior (assessing thoughts and behavior associated with conduct disorder and oppositional defiant behavior) were measured, and each inventory included 20 questions. Every question was scored using a 4-point Likert scale, with 0 indicating never and 3 indicating always. Total scores were summed for each inventory. Thus, higher scores indicated a stronger display of a particular psychological domain. To enable comparison of a child’s score with those of other age-matched children, raw scores were converted (standardized) into T scores (with a mean of 50 and a standard deviation of 10). The internal consistency was indicated as Cronbach’s α coefficients, and ranged from 0.86 to 0.96 for all age groups on all scales, good test-retest reliability was reported to range from 0.74 to 0.93^48^. Validity was supported by correlations with other instruments that assess similar characteristics. Both the reliability and validity were further established using the Chinese version of the BYI-II^43^. All Cronbach’s α coefficients were clearly greater than 0.9 for each of the five inventories, and test-retest reliability ranged from 0.64 to 0.81. Criterion-related validity was also supported for each inventory. Although continuous T scores were generally preferable in statistical modeling, providing greater variability, we wanted to identify children with psychological difficulties above or below the “warning line”, who deserved special attention. We used cutoff points suggested in the BYI-II Manual to categorize students into “lower than normal, normal” and “higher than normal” for each of the five psychological domains of depression, anxiety, anger, disruptive behavior, and self-concept^48,49^.

### Statistical analysis

The sum of urinary inorganic arsenic (iAs^III^ and iAs^V^) and methylated arsenic (MMA^V^ and DMA^V^) concentrations (μg/L) was defined as the total urinary arsenic. The inorganic arsenic percentages (iAs%), MMA^V^%, and DMA^V^% were calculated by dividing the concentration of each species [(iAs^III^ + iAs^V^), MMA^V^ and DMA^V^] by the total urinary arsenic concentrations. The Student’s *t*-test was used to compare differences in the variables of age, biochemical indices, psychological characteristics, and urinary arsenic profiles between elementary school and junior high school students. The χ^2^ test was used to test for differences in categorical variables between elementary school and junior high school students. Analysis of variance (one-way ANOVA) and least significant difference test (post hoc test) with Bonferroni correction were used to compare the differences in variables of urinary arsenic profiles, and HOMA-IR levels among different psychological characteristics. Multiple linear regression models were used to estimate multivariate adjusted associations between the urinary arsenic profiles, HOMA-IR levels and psychological characteristics, and presented in regression coefficients (β) and standard error (SE). For dose-dependent relationships, trend analysis was performed by treating ordinal-score variables as continuous variables in the regression model. For the interaction analysis, the cutoff point for iAs% and HOMA-IR value was the median of the overall students, elementary school students and junior high school students, respectively.

## Electronic supplementary material

Supplementary Table
