# Author Correction: The Methylation Capacity of Arsenic and Insulin Resistance are Associated with Psychological Characteristics in Children and Adolescents

**DOI:** 10.1038/s41598-018-26884-6

**Published:** 2018-06-06

**Authors:** Ying-Chin Lin, Chien-Tien Su, Horng-Sheng Shiue, Wei-Jen Chen, Yi-Hua Chen, Cheuk-Sing Choy, Hung-Yi Chiou, Bor-Cheng Han, Yu-Mei Hsueh

**Affiliations:** 1 Department of Family Medicine, Shung Ho Hospital, Taipei Medical University, Taipei, Taiwan; 2 Department of Health Examination, Wan Fang Hospital, Taipei Medical University, Taipei, Taiwan; 3 Department of Family Medicine, School of Medicine, College of Medicine, Taipei Medical University, Taipei, Taiwan; 4 Department of Family Medicine, Taipei Medical University Hospital, Taipei, Taiwan; 5 School of Public Health, College of Public Health, Taipei Medical University, Taipei, Taiwan; 6 Department of Chinese Medicine, Chang Gung Memorial Hospital, and Chang Gung University College of Medicine, Taoyuan, Taiwan; 7 Emergency Department, Taipei Hospital, Ministry of Health and Welfare, New Taipei City, Taiwan; 8 Department of Medicine, School of Medicine, College of Medicine, Taipei Medical University, Taipei, Taiwan; 9 Department of Public Health, School of Medicine, College of Medicine, Taipei Medical University, Taipei, Taiwan

Correction to: *Scientific Reports* 10.1038/s41598-017-03084-2, published online 08 June 2017

The original version of this Article contained an error in Affiliation 3, which was incorrectly given as ‘Division of Family Medicine, School of Medicine, Taipei Medical University, Taipei, Taiwan’. The correct affiliation is listed below:

Department of Family Medicine, School of Medicine, College of Medicine, Taipei Medical University, Taipei, Taiwan

This error has now been corrected in the HTML and PDF versions of the Article.

