# Supplementary material for: The Methylation Capacity of Arsenic and Insulin Resistance are Associated with Psychological Characteristics in Children and Adolescents
Source: Sci Rep. 2017 Jun 8;7:3094. doi: 10.1038/s41598-017-03084-2 (PMC5465070; doi:10.1038/s41598-017-03084-2)
Supplement: Supplementary file 1 — Supplementary Table [file 41598_2017_3084_MOESM1_ESM.pdf]

The Methylation Capacity of Arsenic and Insulin Resistance are Associated with Psychological Characteristics in Children and Adolescents

Ying-Chin Lin, Chien-Tien Su, Horng-Sheng Shiue, Wei-Jen Chen, Yi-Hua Chen, Cheuk-Sing Choy, Hung-Yi Chiou, Bor-Cheng Han, Yu-Mei Hsueh

Supplementary Table S1. The impact of parents' smoking status on the urinary total arsenic and arsenic methylation capacity indices of children.

Variables	N	Urinary total arsenic (μg/L)	Urinary total arsenic (μg/g creatinine)	iAs%	MMA%	DMA%
Elementary school students						
Father's smoking status						
Non-smoking	135	24.44 ± 1.81	30.17 ± 2.63	4.40 ± 0.30	4.28 ± 0.38	91.32 ± 0.51
Ever smoking	150	24.75 ± 1.81	29.71 ± 2.52	5.15 ± 0.56	5.61 ± 0.52	89.24 ± 0.91
p value		0.90	0.90	0.24	0.04	0.05
Mother's smoking status						
Non-smoking	263	24.16 ± 1.32	29.07 ± 1.80	4.91 ± 0.35	4.88 ± 5.55	90.21 ± 0.57
Ever smoking	22	30.08 ± 5.17	41.86 ± 9.36	3.64 ± 3.83	6.53 ± 1.44	89.83 ± 1.63
p value		0.22	0.19	0.16	0.19	0.85
Junior high school students						
Father's smoking status						
Non-smoking	105	23.56 ± 2.09	22.55 ± 1.90	7.09 ± 0.47	5.57 ± 0.57	87.34 ± 0.79
Ever smoking	199	26.66 ± 1.50	23.42 ± 1.49	7.78 ± 0.41	4.93 ± 0.28	87.28 ± 0.53
p value		0.23	0.72	0.26	0.32	0.95
Mother's smoking status						
Non-smoking	273	26.41 ± 1.37	24.44 ± 1.49	7.59 ± 0.34	5.27 ± 0.28	87.13 ± 0.47
Ever smoking	35	24.87 ± 3.19	19.99 ± 3.51	6.86 ± 0.64	4.72 ± 0.79	88.42 ± 1.17
p value		0.70	0.31	0.31	0.51	0.35

The data regarding father's smoking status were unavailable for eleven elementary school children and 14 junior high school children; mother's smoking status were unavailable for 11 elementary school children and 10 junior high school children.

Supplementary Table S2. The impact of body fat, vegetables and fruit intake of children and family history on the HOMA-IR value in children.

Variables	N	HOMA-IR value	Variables	N	HOMA-IR value
Elementary school students			*Junior high school students*		
Body fat[a]			Body fat[a]		
Non-obesity	155	1.58 ± 0.11	Non-obesity	253	3.35 ± 0.19
Obesity	141	4.14 ± 0.64	Obesity	65	6.14 ± 0.62
p value		< 0.01	*p* value		< 0.01
Number of vegetables intake (per week)			Number of vegetables intake (per week)		
1-5	28	4.09 ± 1.28	1-5	41	4.00 ± 0.75
6-10	65	2.90 ± 0.90	6-10	70	4.64 ± 0.63
11-15	109	2.23 ± 0.19	11-15	95	3.48 ± 0.21
> 16	84	2.97 ± 0.71	> 16	111	3.73 ± 0.27
p value		0.42	*p* value		0.22
Number of fruit intake (per week)			Number of fruit intake (per week)		
1-5	55	2.89 ± 0.40	1-5	80	4.17 ± 0.53
6-10	116	2.65 ± 0.52	6-10	108	4.04 ± 0.39
11-15	58	3.08 ± 0.98	11-15	75	3.89 ± 0.33
> 16	54	2.66 ± 0.70	> 16	54	3.40 ± 0.22
p value		0.96	*p* value		0.67
Father's diabetes histories			Father's diabetes histories		
No	182	2.72 ± 0.38	No	220	3.86 ± 0.25
Yes	88	2.83 ± 0.69	Yes	79	3.88 ± 0.32
p value		0.89	*p* value		0.97
Father's hypertension histories			Father's hypertension histories		
No	162	2.91 ± 0.44	No	191	3.98 ± 0.25
Yes	108	2.52 ± 0.53	Yes	108	3.67 ± 0.35
p value		0.58	*p* value		0.47
Mother's diabetes histories			Mother's diabetes histories		
No	283	2.81 ± 0.33	No	304	3.96 ± 0.22
Yes	2	4.40 ± 3.55	Yes	5	2.80 ± 0.37
p value		0.69	*p* value		0.03
Mother's hypertension histories			Mother's hypertension histories		
No	272	2.80 ± 0.34	No	294	3.91 ± 0.20
Yes	13	3.43 ± 0.90	Yes	15	4.57 ± 2.10
p value		0.52	*p* value		0.76

[a]Body fat more than 25% in male or more than 30% in female were defined as obesity.

The data regarding number of vegetables intake were unavailable for eleven children; number of fruit intake were unavailable for 14 children; father's diabetes and hypertension histories were unavailable for 45 children; mother's diabetes and hypertension histories were unavailable for 20 children.
